# Zanubrutinib‑Induced Symmetrical Soft Tissue Forearm Swelling in a Patient With Waldenström Macroglobulinemia

**DOI:** 10.7759/cureus.92366

**Published:** 2025-09-15

**Authors:** Victor M Samperio, Aishwarya Ghonge, Constantin A Dasanu

**Affiliations:** 1 Department of Medicine, Eisenhower Medical Center, Rancho Mirage, USA; 2 Department of Oncology and Hematology, Lucy Curci Cancer Center, Eisenhower Health, Rancho Mirage, USA

**Keywords:** bruton tyrosine kinase (btk) inhibitor, musculoskeletal toxicity, soft tissue swelling, waldenström macroglobulinemia, zanubrutinib

## Abstract

Zanubrutinib is a second-generation Bruton tyrosine kinase (BTK) inhibitor approved for the treatment of several indolent B-cell lymphomas. Although it has a more favorable safety profile than its predecessor, ibrutinib, rare adverse effects continue to emerge with broader clinical use. We report the case of an 87-year-old man with Waldenström macroglobulinemia (WM) who developed bilateral, non-pitting, soft tissue swelling of the forearms approximately 16 weeks after initiating zanubrutinib 160 mg orally twice daily. The swelling extended from the elbows to the hands, was symmetrical, non-tender, and not associated with any other symptoms. Doppler ultrasound ruled out deep vein thrombosis, and no alternative causes such as trauma, infection, or hypoalbuminemia were identified. A 50% dose reduction of zanubrutinib led to partial improvement, while complete resolution was observed 12 weeks after drug discontinuation. Unlike previously reported cases of BTK inhibitor-associated edema, which typically involve the lower extremities and present unilaterally or with associated skin changes, this case is unique in its bilateral presentation and upper extremity location without cutaneous involvement. The mechanism remains unclear but may involve vascular endothelial dysfunction or altered lymphatic drainage. Prompt recognition of this atypical presentation is essential for avoiding unnecessary interventions and optimizing therapeutic decisions.

## Introduction

Based on robust clinical trial data, zanubrutinib and acalabrutinib have demonstrated durable responses and reduced toxicity in several hematologic malignancies compared to the first-generation Bruton tyrosine kinase (BTK) inhibitor ibrutinib. BTK inhibitors irreversibly block Bruton’s tyrosine kinase, a critical mediator of B-cell receptor and other immune signaling pathways, thereby impairing malignant B-cell activation and survival. While their cardiovascular risk is low, real-world surveillance has identified several cutaneous and vascular side effects [1-4]. We report a unique pattern of non-pitting soft tissue forearm swelling in a patient receiving zanubrutinib for Waldenström macroglobulinemia (WM).

## Case presentation

An 87-year-old Caucasian man without significant cardiovascular or liver disease was diagnosed with WM in August 2024. His prior medical history included early-stage cutaneous angiosarcoma of the left superior helical rim, multiple basal cell carcinomas (BCCs) and cutaneous squamous cell carcinomas (cSCCs) treated with local excisional therapy (face, ear, upper extremities, and torso), and radiation therapy for right forearm cSCC. He underwent extensive workup due to a persistent normocytic anemia, and a monoclonal gammopathy IgM kappa (0.97 g/dL) with kappa free light chain elevation was found.

A bone marrow study in August 2024 showed a B-cell lymphoproliferative disorder with a kappa-restricted plasma cell population (40-50%). Cytogenetics and next-generation sequencing revealed a -Y abnormality and the presence of the MYD88 L265P mutation. Based on these findings, the diagnosis of WM was made, and zanubrutinib 160 mg twice a day was initiated. In November 2024, the patient developed an erythematous rash over the abdomen and chest, which resolved with over-the-counter hydrocortisone 1% cream twice a day.

**Figure 1:**
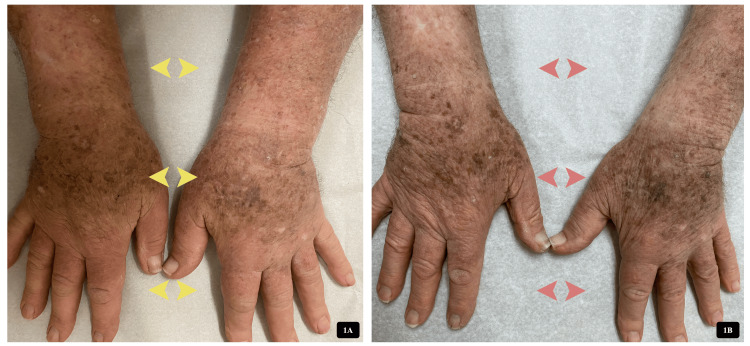
Zanubrutinib-induced symmetrical forearm and hand soft tissue swelling in the index patient. (A) Initial presentation showing bilateral, non-pitting, indurated soft tissue swelling of the forearms and hands (yellow arrows). (B) Improvement in soft tissue swelling (red arrows) four weeks after discontinuation of zanubrutinib.

At that time, a significant reduction in M-protein was noted (0.48 g/dL), consistent with a partial response to zanubrutinib. By December 2024, he developed bilateral forearm, non-tender, non-pitting, indurated soft tissue swelling that began immediately below the elbow level and gradually progressed in a caudal trajectory until it involved both hands (Figure 1A). The patient reported heaviness in both upper extremities, but there were no systemic symptoms (e.g., fever, chills), no clinical evidence of cellulitis, joint deformity, erythema, or leukocytosis. Upper extremity Doppler ultrasound excluded deep venous thrombosis. Albumin was within normal limits. No new medications or trauma to the upper extremities were reported.

**Figure 2:**
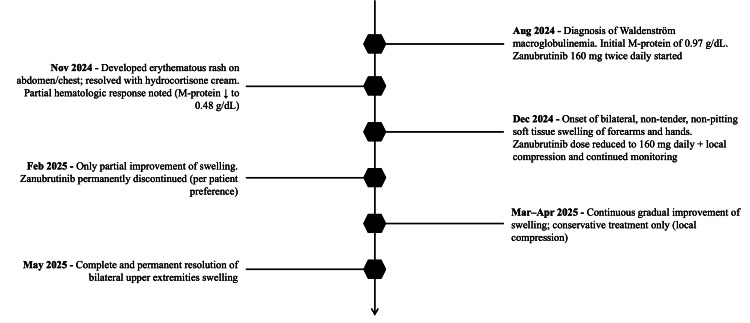
Clinical timeline of events Timeline summarizing the patient’s clinical course, including time of diagnosis, initiation of zanubrutinib, onset of adverse events, management, and adverse event resolution.

Initial management included a reduction of zanubrutinib to 160 mg daily, local compression, and continued outpatient monitoring. However, due to only partial improvement of the swelling (Figure 1B), and per the patient’s request, zanubrutinib was permanently discontinued in February 2025. By May 2025, the soft tissue swelling had resolved completely, and the patient has remained symptom-free since. The temporal relationship between zanubrutinib initiation, adverse event onset, dose modification, discontinuation, and resolution is illustrated in Figure 2.

Given the absence of alternative etiologies, the temporal relationship between starting the drug and developing the symptoms, and the improvements seen after stopping the drug, the likelihood of zanubrutinib being the causal agent became stronger. The Naranjo Adverse Drug Reaction Probability Scale also supports this hypothesis, yielding a score of 7, which indicates a “probable” adverse drug reaction (Table 1).

**Table 1:** The Naranjo adverse drug reaction (ADR) probability scale questionnaire. The Naranjo Adverse Drug Reaction Probability Scale is a validated, questionnaire-based algorithm designed to estimate the likelihood of a causal relationship between a drug and an observed adverse event. It incorporates weighted criteria including temporal correlation, alternative etiologies, drug concentration data, dose-response relationships, and prior patient experience. Based on the cumulative score, the adverse event is classified as definite (≥9), probable (5–8), possible (1–4), or doubtful (0). The assessment is performed on an individual drug basis and does not account for pharmacologic interactions; the presence of confounding factors may reduce the assigned probability of causality.

To assess the adverse drug reaction, please answer the following questionnaire and give the pertinent score	Yes	No	Do not know	Score
1. Are there previous conclusive reports on this reaction?	+1	0	0	+1
2. Did the adverse event appear after the suspected drug was given?	+2	-1	0	+2
3. Did the adverse reaction improve when the drug was discontinued or a specific antagonist was given?	+1	0	0	+1
4. Did the adverse event reappear when the drug was re-administered?	+2	-1	0	0
5. Are there alternative causes that could have caused the reaction?	-1	+2	0	+2
6. Did the reaction reappear when a placebo was given?	-1	+1	0	0
7. Was the drug detected in blood (or other fluids) at toxic concentrations?	+1	0	0	0
8. Was the reaction worsened when the dose was increased or lessened when dose was decreased?	+1	0	0	0
9. Did the patient have a similar reaction to the same or similar drugs in any previous exposure?	+1	0	0	0
10. Was the adverse event confirmed by any objective evidence?	+1	0	0	+1
			Total	7

## Discussion

Zanubrutinib is a second-generation irreversible BTK inhibitor engineered to maximize target selectivity and minimize off-target kinase inhibition. It is currently approved by the United States (US) Food and Drug Administration (FDA) for WM, mantle cell lymphoma, marginal zone lymphoma, chronic lymphocytic leukemia/small lymphocytic lymphoma, and relapsed/refractory follicular lymphoma in combination with obinutuzumab.

In the ASPEN trial of zanubrutinib versus ibrutinib in WM, peripheral edema was reported in 9% of zanubrutinib-treated patients compared with 19% of those receiving ibrutinib [1]. In the MAGNOLIA trial of relapsed/refractory marginal zone lymphoma, peripheral edema was not highlighted among the commonly reported adverse events [2]. Rare reports of zanubrutinib-induced peripheral edema have involved the lower extremities, often associated with concomitant cutaneous and/or articular findings [5,6]. However, symmetrical soft tissue swelling has not previously been documented as a drug-related toxicity of zanubrutinib or other BTK inhibitors.

The pathophysiology of BTK inhibitor-induced edema is unclear but may involve a combination of vascular endothelial dysfunction and altered lymphatic drainage. Off-target effects have been reported with both ibrutinib and zanubrutinib. An alternative hypothesis involves localized capillary leak phenomena, potentially exacerbated by underlying lymphatic architectural disruption from prior therapy or malignancy [6,7].

In contrast to other cases of BTK inhibitor-associated peripheral edema, our case did not present with concomitant erythematous plaques, arthralgias, pitting edema, telangiectasias, macules, or unilaterality, but rather as non-tender, non-pitting, indurated, bilateral soft tissue swelling. The absence of systemic causes, resolution after drug withdrawal, and the Naranjo score of 7 support a probable causal association (Table 1) [8]. Re-administration of zanubrutinib (re-challenge) was not pursued due to ethical considerations and the patient’s preference.

## Conclusions

Early recognition of symmetrical soft tissue swelling as a potential drug reaction is critical for timely diagnosis, symptom control, and avoidance of unnecessary interventions. Clinicians should consider zanubrutinib as a possible etiology in patients presenting with unexplained upper extremity soft tissue swelling during therapy, especially in populations with a history of potential insult to the angiolymphatic system, such as older age, nonmelanoma skin cancers, and prior radiation therapy. Conservative management, with dose reduction or drug withdrawal, seems to be sufficient in most cases.

Further pharmacovigilance studies and mechanistic investigations are needed to better characterize the vascular, soft tissue, articular, and immunologic sequelae of BTK inhibition. Until then, clinicians should maintain a high index of suspicion for rare but reversible zanubrutinib-associated adverse events.

## References

[1] Tam CS, Opat S, D'Sa S (2020). A randomized phase 3 trial of zanubrutinib vs ibrutinib in symptomatic Waldenström macroglobulinemia: the ASPEN study. Blood.

[2] Opat S, Tedeschi A, Linton K (2021). The MAGNOLIA trial: zanubrutinib, a next-generation Bruton tyrosine kinase inhibitor, demonstrates safety and efficacy in relapsed/refractory marginal zone lymphoma. Clin Cancer Res.

[3] Rogers KA, Thompson PA, Allan JN (2021). Phase II study of acalabrutinib in ibrutinib-intolerant patients with relapsed/refractory chronic lymphocytic leukemia. Haematologica.

[4] Byrd JC, Hillmen P, Ghia P (2021). Acalabrutinib versus ibrutinib in previously treated chronic lymphocytic leukemia: results of the first randomized Phase III trial. J Clin Oncol.

[5] Dasanu CA (2019). Severe arthritic syndrome due to ibrutinib use for chronic lymphocytic leukemia. J Oncol Pharm Pract.

[6] Meledathu S, Chu P, Feldman T, Goy AH, Rosenstein RK (2024). Bruton's tyrosine kinase inhibitor associated localized extremity edema and erythema. JAAD Case Rep.

[7] Javidi-Sharifi N, Brown JR (2024). Evaluating zanubrutinib for the treatment of adults with chronic lymphocytic leukemia or small lymphocytic lymphoma. Expert Rev Hematol.

[8] Naranjo CA, Busto U, Sellers EM (1981). A method for estimating the probability of adverse drug reactions. Clin Pharmacol Ther.

